# Role of Conservative Management in Stump Appendicitis: A Case Series

**DOI:** 10.31729/jnma.7578

**Published:** 2022-09-30

**Authors:** Nabin Paudyal, Fathmath Asra Saeed, Bijaya Shrestha

**Affiliations:** 1 Department of General and Laparoscopic Surgery, Nobel Medical College Teaching Hospital, Biratnagar, Morang, Nepal; 2 Department of Radiology, Nobel Medical College Teaching Hospital, Biratnagar, Morang, Nepal

**Keywords:** *appendectomy*, *appendicitis*, *case reports*

## Abstract

Stump appendicitis is a rare, delayed complication of appendectomy. It is seen following both open and laparoscopic appendectomy and may occur weeks to years following the initial appendectomy. We report two cases of stump appendicitis seen at our hospital. Both cases were diagnosed based on radiological findings and successfully managed conservatively with antibiotics. Although the usually recommended treatment for stump appendicitis is completion appendectomy, conservative management may be suitable for some patients. This report highlights the possibility of utilizing a conservative approach in the management of stump appendicitis compared to the recommended operative intervention. Awareness of the possibility of stump appendicitis is crucial for early diagnosis and treatment, to prevent potentially catastrophic complications.

## INTRODUCTION

Stump appendicitis is a rare, long-term complication of appendectomy with a reported incidence of 1 in 50,000.^1^ The residual stump left after an initial appendectomy may develop recurrent inflammation, producing symptoms similar to acute appendicitis. However, this diagnosis is often not considered in patients with a prior history of appendectomy.^2^ Failure to identify this condition may lead to increased morbidity and hospital stay as well as complications such as perforation, abscess formation, and sepsis.

Here, we report two cases of stump appendicitis that presented to our emergency department with abdominal pain, varying durations following an appendectomy. Both patients were treated conservatively with antibiotics and made an uneventful recovery.

## CASE REPORTS

### CASE 1

A 41-year-old gentleman presented to the emergency department with abdominal pain in the right lower quadrant for 3 days. There was no history of fever, vomiting, altered bowel habits or urinary symptoms. He had undergone an uneventful appendectomy 2 years ago at our centre after presenting with similar symptoms. Family history and socioeconomic history were unremarkable. On admission, he was afebrile and his vitals were stable. Abdominal examination revealed tenderness in the right iliac fossa. His laboratory tests were normal.

**Figure 1:**
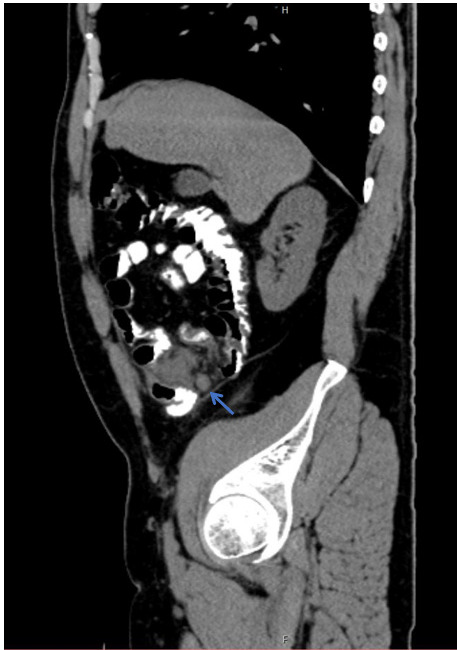
CECT (Contrast Enhanced Computed Tomography) in sagittal view showing blind-ending tubular structure arising from the caecal pole with adjacent fat stranding.

**Figure 2:**
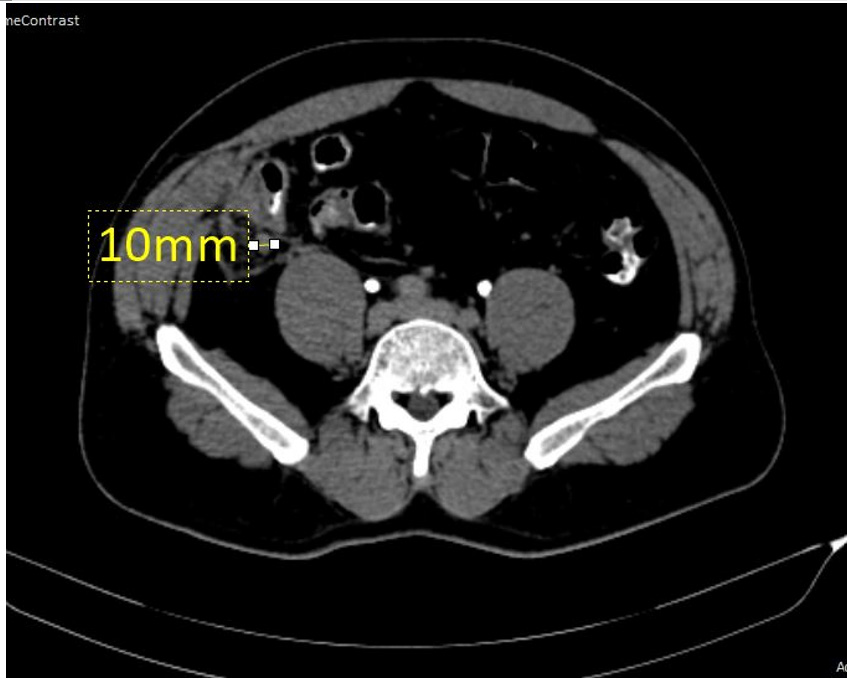
CECT axial view showing a tubular structure (10 mm in diameter) extending from the base of the caecum with thickened and enhancing walls, stranding of the adjacent fat.

**Figure 3:**
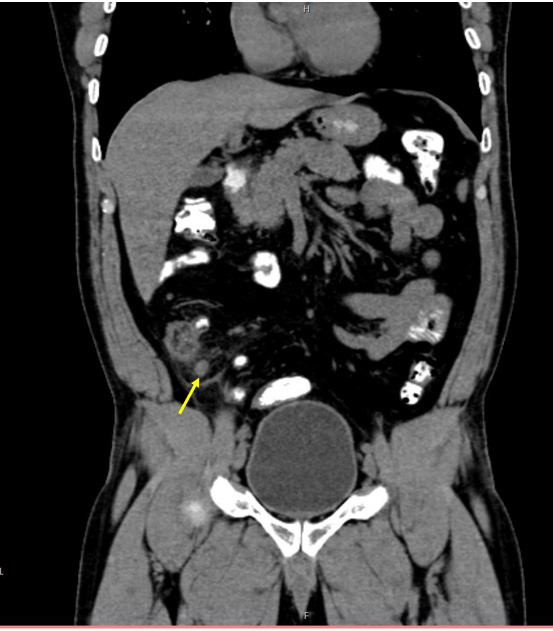
CECT coronal view showing periapical inflammatory change, as well as caecal wall thickening.

As a part of routine investigation abdominal ultrasonography (USG) was advised which revealed an appendicular stump with surrounding periappendiceal fluid collection. An abdominal Computed Tomography (CT) scan revealed inflammatory changes in the region of the cecum with adjacent fat stranding and lymphadenopathy (Figure 1-3). Hence the diagnosis of stump appendicitis was made based on radiological findings and a history of appendectomy.

The patient was admitted to the general surgery ward and started on IV (Intravenous) antibiotics (cefoperazone one gram two times a day and metronidazole 500 mg three times a day for 5 days). He was kept nil per oral for 5 days and was managed with analgesics and antiemetics as well. He made an uneventful recovery and was discharged after 6 days with 1 week of oral antibiotics (cefixime 200 mg orally two times a day and metronidazole 400 mg orally three times a day). He did not have any difficulties at 2 weeks follow-up. Follow-up USG reports showed resolution of the inflamed stump.

### CASE 2

**Figure 4:**
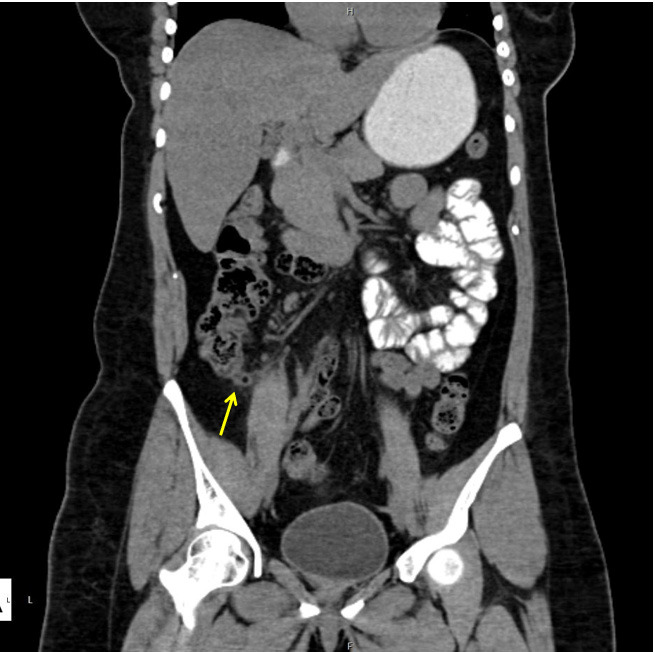
CECT coronal view showing a tip of the remnant appendix tissue arising from the caecal wall.

**Figure 5:**
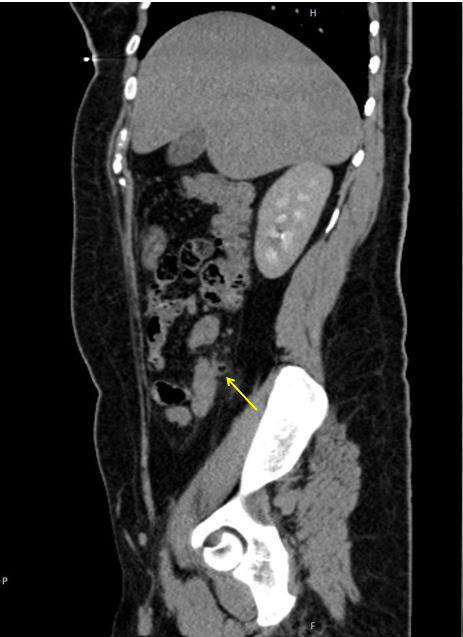
CECT sagittal view showing air foci within the tubular structure with minimal surrounding fat stranding.

A 23-year-old lady with a previous history of laparoscopic appendectomy 1 month back, presented to our emergency department after 2 days of rightsided abdominal pain, fever, nausea, and vomiting. The pain was initially dull aching in character, worsened gradually and was associated with decreased appetite. She denied having altered bowel habits or urinary symptoms. Her last menstrual period was 10 days before the presentation. On physical examination, vitals were stable. On abdominal examination, tenderness was present in the right iliac fossa. Rebound tenderness was present. Inflammatory markers were not raised. As a part of routine investigation, abdominal ultrasonography (USG) was advised which showed a small stump appendix with surrounding inflammatory changes and fluid collection. Abdominal CT was done which supported the USG findings (Figure 4-5).

A stump of the appendix measuring 8 mm with an air dock within the lumen was visualised along with inflammatory changes. A conservative approach with IV antibiotics was prescribed similar to case 1. Supportive treatment was given and the patient improved remarkably. She was discharged after a 7 day course of antibiotic therapy with oral antibiotics similar to case 1. She remained asymptotic for 1 month following discharge.

## DISCUSSION

Stump appendicitis is defined as the interval repeated inflammation of remaining residual appendiceal tissue after an appendectomy, either due to impaction of a fecolith or secondary to an ischemic process.^3^ Rose, in 1945, was the first to describe stump appendicitis in two patients who had previously undergone appendectomy for acute appendicitis.^4^ It is a rare occurrence with a reported incidence of 1 in 50000 cases but the real incidence is expected to be much higher as this condition is often underestimated.^4^ The incidence has been increasing recently which may be attributed to increased awareness among treating physicians as well as the easy availability of CT scans.

The laparoscopic technique has been implicated as a factor in the recent increase in the incidence of stump appendicitis.^5^ Lack of a three-dimensional perspective and the absence of tactile feedback may obscure the base of the appendix, leaving behind a longer appendicular stump which may act as a reservoir for fecoliths and predispose to the development of stump appendicitis. Although a majority of reported cases have occurred following an open appendectomy, it may not accurately reflect the incidence in comparison to the laparoscopic technique, as the latter is relatively newer to the traditionally practised open method.^5^

The time interval between initial operation and recurrence of symptoms could range from 2 weeks to years after appendectomy. It has been suggested that this duration may be significantly shorter following laparoscopic appendectomy.^6^ In our cases, stump appendicitis developed just 1 month following a laparoscopic appendectomy in contrast to 2 years after an open appendectomy.

Clinical presentation of stump appendicitis is similar to acute appendicitis. Common symptoms include abdominal pain, usually in the right iliac fossa associated with nausea and vomiting.^7^ These nonspecific symptoms along with a past history of appendectomy make the diagnosis challenging. Hence, radiological examinations are of great significance in diagnosing this uncommon phenomenon. An abdominal CT scan is considered the gold standard and shows findings similar to acute appendicitis including pericecal inflammation, cecal wall thickening, free or loculated fluid in the right paracolic gutter, and infiltration of the surrounding fat. A long appendiceal stump might be visualised as a tubular, thick-walled, fluid-filled, enhancing structure.^8^ In both our cases, simple abdominal ultrasound was able to suspect the condition accurately and was further strengthened by typical CT scan findings.

Completion appendectomy, either by laparotomy or laparoscopy has been considered the treatment of choice and is generally sufficient when the base of the appendix can be identified and the cecum is not significantly inflamed.^9^ More extensive surgery such as ileocecectomy and right hemicolectomy may be required in some cases. Conservative treatment with antibiotics is proving to be effective in some instances, especially in the absence of appendicoliths or evidence of perforation, as seen in our cases.^10^ Careful follow-up is mandatory in these cases to prevent the recurrence of chronic appendicitis.

Stump appendicitis is a rare but serious complication of appendectomy, whose incidence has been increasing in recent years. As it presents with vague, nonspecific symptoms, a high index of suspicion is required for diagnosing the condition. Imaging techniques like USG or CT scan may aid in establishing the diagnosis. Although stump resection is the preferred treatment in most reported cases, conservative management with antibiotics may be an effective, alternative treatment option.
